# Isolated Double-Positive Optic Neuritis: A Case of Aquaporin-4 and Myelin Oligodendrocyte Glycoprotein Antibody Seropositivity

**DOI:** 10.7759/cureus.15389

**Published:** 2021-06-02

**Authors:** Matthew C Mason, Dario A Marotta, Hassan Kesserwani

**Affiliations:** 1 Department of Research, Alabama College of Osteopathic Medicine, Dothan, USA; 2 Department of Neurology, Division of Neuropsychology, University of Alabama, Birmingham, USA; 3 Neurology, Flowers Medical Group, Dothan, USA

**Keywords:** double positive optic neuritis, optic neuritis, neuromyelitis optica spectrum disorder, myelin-oligodendrocyte glycoprotein (mog), aquaporin-4 antibody, myelin-oligodendrocyte glycoprotein antibody disease

## Abstract

Optic neuritis (ON) causes acute vision loss with typical and atypical profiles, serological markers, imaging findings, and clinical outcomes depending on the associated underlying pathophysiology. Neuromyelitis optica (NMO) and myelin oligodendrocyte glycoprotein antibody disease (MOGAD) are the usual causes of acute severe sequential or simultaneous bilateral optic neuritis. These conditions are usually accompanied by multi-level spinal cord demyelination, and notably, they are typically positive for either NMO or Myelin oligodendrocyte glycoprotein (MOG) autoantibodies, but rarely both. We present a case of isolated sequential bilateral optic neuritis that was seropositive for both NMO and MOG antibodies.

## Introduction

Optic neuritis (ON) is usually a demyelinating inflammatory injury to one or both optic nerves. The typical clinical presentation of optic neuritis is unilateral acute vision loss with associated retrobulbar pain. In contrast, atypical presentations of ON have more severe vision loss that is rapidly sequential or simultaneous bilateral vision loss, steroid-resistant, and may portend a worse outcome if untreated [1]. These fundamental differences led to identifying distinct clinical conditions known as neuromyelitis optica (NMO) and myelin oligodendrocyte glycoprotein antibody disease (MOGAD).

Typical ON is a condition occurring in 1.5-5.1 cases per 100,000 with a higher incidence in females, whereas NMO and MOGAD are 0.5-1.4 per 100,000 persons [2-4]. NMO, a variant of NMO spectrum disorder (NMOSD), and MOGAD are characterized by serological positivity to aquaporin-4 (AQP-4) antibody and MOG antibody for NMO/NMOSD and MOGAD, respectively, but the frequency of double seropositivity has rarely been reported [5-7]. Here we report a 66-year-old female with steroid-responsive isolated sequential bilateral optic neuritis and seropositivity to both NMO and MOG antibodies.

## Case presentation

A 66-year-old female presented to the neurology clinic sequentially bilateral vision loss with associated retrobulbar pain on extraocular movement. The patient reports that she experienced acute onset left eye vision loss and pain that occurred without an inciting event eight weeks prior. Following initial improvement with an oral five-day tapered 48mg methylprednisolone pack, she developed acute onset right eye vision loss and pain. She denied any headache, numbness, weakness, vertigo, ataxia, diplopia, fatigue, urogenital dysfunction, or slurred speech, cough, shortness of breath, rashes, conjunctival erythema, and arthralgias. She also denied the Lhermitte phenomenon.

The patient reported a past medical history of hypertension and an episode of Parsonage-Turner syndrome 15 years before presentation. Her medications included amlodipine, hydrochlorothiazide, and low dose aspirin. She denied any remote or recent history of tobacco, alcohol, and illicit drug use. Family history was also unremarkable, and she denied a family history of underlying autoimmune disease.

**Figure 1:**
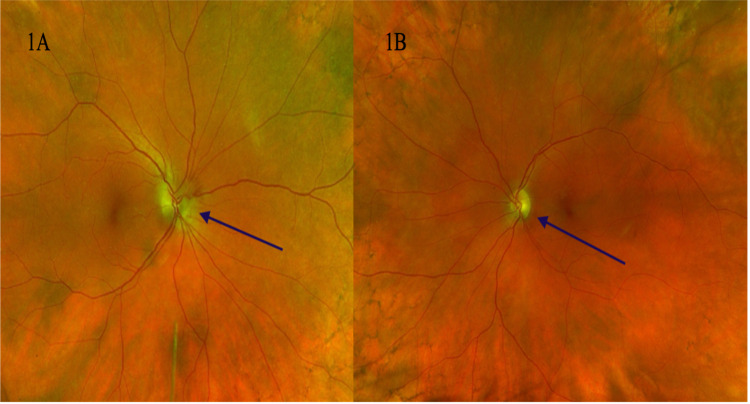
1A: Left eye fundus, optic disc edema (blue arrow). 1B: Right eye fundus, optic disc edema (blue arrow)

The patient is 5 feet 6 inches, weighing 176 pounds, with a body mass index of 28.4kg/m^2^. Her vitals revealed a blood pressure of 142/88 mmHg, heart rate of 86 beats per minute, and oxygen saturation of 97%. Physical exam revealed normal gait stance, cadence and tandem. Dilated fundoscopic exam revealed bilateral papilledema (Figure 1).

 

**Figure 2:**
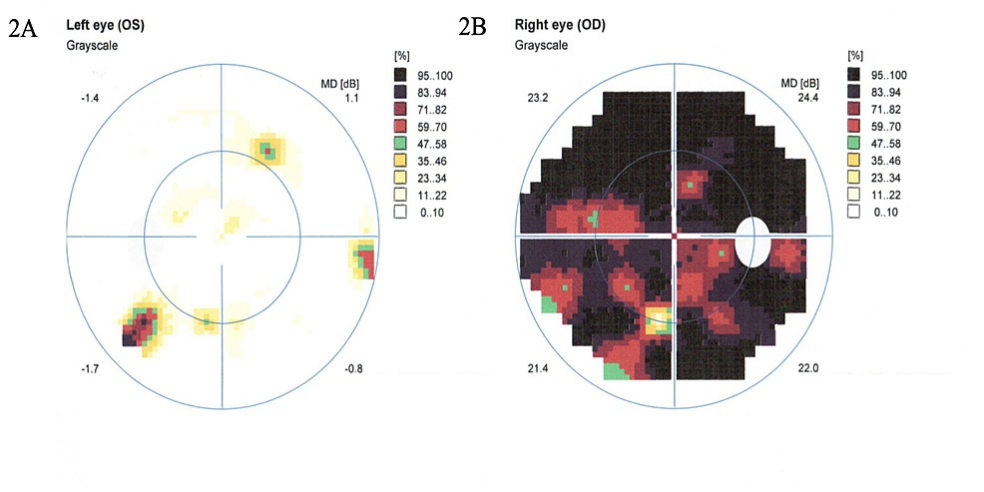
2A: Left eye Humphrey visual field testing showing constricted visual field. 2B: Right eye Humphrey visual field testing showing near blackout of vision. Decibel (dB).

Her visual acuity in the right eye was limited to counting fingers, while left eye visual acuity was 20/40. Humphrey visual field testing is shown in figure 2.

**Figure 3:**
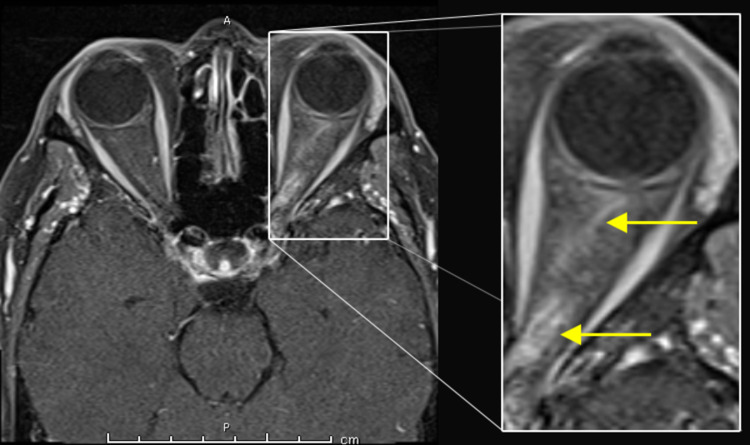
T1-weighted axial MRI gadolinium-enhanced image: enhancing and thickened optic nerve (yellow arrows)

The extra-ocular motion was complete in all directions. The right eye demonstrated a Marcus-Gunn pupil. Consensual light and accommodative reflexes were intact. No ptosis was noted. Right eye visual acuity was greater than 20/200 (count fingers), left eye visual acuity was 20/40. The remaining cranial nerves were normal. Muscle strength testing in all extremities was 5/5 on the medical research council grading scale. Coordination with finger-to-nose and heel-to-shin testing was normal bilaterally. Deep tendon reflexes were 2+ bilaterally in the upper and lower extremities. Sensory examination to touch-pressure, pin-prick, vibration and joint-position sense was normal in the fingers and toes. Magnetic resonance imaging (MRI) of the brain and orbits revealed enhancement of the left optic nerve with no cerebral white matter plaques (figure 3). 

Carotid artery duplex ultrasound of the neck revealed anterograde flow with mild, subclinical intimal thickening without significant plaque, occlusion, or stenosis. A visual evoked potential study revealed bilateral anterior visual conduction deficits with prolonged P100 latencies bilaterally, worse in the right eye. Lumbar puncture with cerebral spinal fluid and serology analyses was conducted and can be seen below in Table 1. Most notably, serum NMO and MOG antibodies were positive, while cerebral spinal fluid (CSF) markers, including CSF oligoclonal bands and intrathecal synthesis of immunoglobulins, were negative.

**Table 1:** Results of CSF and serological analyses CSF = Cerebral spinal fluid, N/A = Not applicable, RBC = Red blood cells, IgG = Immunoglobulin G, ANA = Antinuclear antibody, RPR = Rapid plasma reagin, ANCA = Antineutrophil cytoplasmic antibody

TEST	RESULT	INTERPRETATION	REFERENCE RANGE	UNITS
CSF				
Color	Colorless	Normal	Colorless	N/A
Clarity	Clear	Normal	Clear	N/A
Total protein	33.4	Normal	0.0-44.0	mg/dL
Glucose	58	Normal	40-70	mg/dL
Nucleated cell	1	Normal	0-5	cells/microliter
RBC	0	Normal	None seen	cells/microliter
NMO/AQP4 IgG	<1.1	Normal	<1.1	N/A
Oligoclonal bands	0	Normal	<4	N/A
SERUM				
NMO IgG Autoantibodies	3.4	Positive	0.0-3.0	U/mL
MOG antibodies	1:100	Positive	<1:10	Titer
ANA	1:40	Borderline	<1:40	Titer
Anti-centromere antibodies	<1:40	Normal	<1:40	Titer
RPR	Non-reactive	Normal	Non-reactive	N/A
Anti-myeloperoxidase antibodies	<9.0	Normal	0.0-9.0	U/mL
Anti-proteinase-3 antibodies	<3.5	Normal	0.0-3.5	U/mL
Cytoplasmic ANCA	<1:20	Normal	<1:20	Titer
Perinuclear ANCA	<1:20	Normal	<1:20	Titer
Atypical perinuclear ANCA	<1:20	Normal	<1:20	Titer

Our patient’s treatment course included an oral five-day 48mg tapered dose methylprednisolone pack following her initial onset of unilateral left eye vision loss, which surprisingly led to significant improvement in visual acuity. Upon developing the right eye vision loss, she was scheduled to receive the standard steroid therapy with a three-day regimen of daily one-gram intravenous methylprednisolone (IVMP). However, the patient could not tolerate IV therapy after one dose and was restarted on oral prednisone at a dose of 20mg twice daily. The response by the seventh day of treatment was dramatic, and she was able to read messages on her cell phone with her right eye. The plan is to taper the steroid dose by 10mg every month slowly.

## Discussion

In recent years, atypical, clinically overlapping presentations of optic neuritis and spinal cord syndromes in combination with varying CSF and serology findings have led to the distinction of NMO, NMOSD, and MOGAD. The separation of these syndromes was further supported by discovering NMO and MOG antibodies with a relatively high degree of sensitivity and specificity. Modern serological testing for NMO have sensitivities of 75% and specificities that approach 100% [8]. It was proposed in the literature that these antibodies played independent roles in the pathophysiological development of NMO and MOGAD because the incidence of double seropositive NMO and MOG antibodies were not or rarely reported [6,9]. A review of the literature for double seropositive optic neuritis cases can be visualized below in Table 2.

**Table 2:** Reported cases of serological double positivity to NMO and MOG antibody

Study	Number of Cases
dos Passos et al. [5]	13
Ishikawa et al. [10]	1
Woodhall et al. [11]	1
Matsuda et al. [12]	2

In 2015, international expert consensus outlined the diagnostic criteria for NMO, which consisted of 1) At least one core clinical characteristic (optic neuritis, transverse myelitis, area postrema syndrome, acute brainstem syndrome, symptomatic narcolepsy or acute diencephalic clinical syndrome with NMOSD-typical diencephalic MRI lesions); 2) Positive serology for AQP4-Immunoglobulin G (IgG); 3) Exclusion of alternative diagnoses [13]. In 2018, international expert consensus described the diagnostic criteria for MOGAD, which included: 1) Monophasic or relapsing acute ON, myelitis, brainstem encephalitis, or encephalitis, or any combination of these syndromes; 2) MRI or electrophysiological (visual evoked potentials in patients with isolated ON) findings compatible with CNS demyelination; 3) Seropositivity for MOG-IgG [9]. Below, we compare and contrast the findings in NMO and MOGAD (Table 3).

**Table 3:** Comparison of NMO and MOGAD characteristics [14] ADEM = Acute disseminated encephalomyelitis, MDEM = Multiphasic disseminated encephalomyelitis, CSF- Cerebrospinal fluid, MOG- Myelin oligodendrocyte glycoprotein, IgG- Immunoglobulin G, NMO- Neuromyelitis optica, MOGAD- myelin oligodendrocyte glycoprotein antibody disease, AQP-4- Aquaporin-4

Characteristic	NMO	MOGAD
Age at onset	~40	~20-30
Incidence (per 100,000)	0.5	0.2-1.4
Serological antibodies	AQP-4 IgG	MOG IgG
CSF	Oligoclonal bands in 5-10%, mononuclear pleocytosis	Oligoclonal bands in 40%, mononuclear pleocytosis
Female:male ratio	1-2:1	8-9:1
Clinical presentation	Optic neuritis, transverse myelitis, area postrema syndrome, brainstem syndrome, narcolepsy or acute diencephalic syndrome, cerebral syndrome with NMOSD-typical brain lesions	ADEM-like (ADEM, MDEM, ADEM optic neuritis, encephalitis) or opticospinal (optic neuritis, myelitis) or brainstem encephalitis
Optic neuritis (laterality, severity, and outcome)	Bilateral more often than unilateral, often posterior optic pathway, involvement of optic chiasma, long lesion, often recurrent, severe, often residual deficits	Bilateral more often than unilateral, often anterior optic pathway, long lesion, often recurrent, severe, good recovery
MRI findings (brain)	Peri-ependymal lesions surrounding the ventricular system. Lesions involving corticospinal tracts, or no brain lesions	ADEM-like brain lesions, or no brain lesions, long-segment spinal lesions
MRI findings (spine)	Long-segment lesions (>3 vertebral segments); typically involving cervicothoracic segment; central cord predominance	Long-segment lesions (>3 vertebral segments); typically involving thoracolumbar segment and conus; confined to grey matter
Clinical course	Relapsing	Monophasic or relapsing

We present a case of double seropositive sequential bilateral ON. This presentation is sporadic due to the double seropositivity itself, but even more so because of the late age of onset, isolated ON without clinical signs of acute myelitis and area postrema syndrome, and lack of characteristic MRI findings. Additionally, our case can reasonably be diagnosed using both NMO and MOGAD expert consensus diagnostic criteria despite these conditions having different antigen targets and unique underlying pathophysiology. Intravenous steroid therapy has been the gold standard treatment for ON with potential add-on therapies with plasma exchange therapy, T-cell modulators, and B-cell modulators for ON cases with steroid resistance or recurrence [15,16]. In a retrospective review of 70 patients with MOGAD on maintenance therapy, intravenous immunoglobulin was associated with the lowest median annualized relapse rate, followed by azathioprine, rituximab and mycophenolate mofetil [17]. Our patient obtained notable improvement in visual acuity with oral steroid therapy and continued oral therapy following the onset of bilateral ON as she could not tolerate IV therapy due to cumbersome side effects. 

Upon reviewing the literature, there is an apparent paucity of patients with double seropositivity to NMO and MOGAD antibodies. The clinical outcomes for NMO and MOGAD are well established. A diagnosis of NMO with positivity to AQP-4 antibody portends a much higher relapse rate with an unfavourable outcome compared to NMO with negative AQP-4 antibody or isolated MOGAD. However, the prognosis of double positivity ON is unknown. While our patient responded quite well to steroid therapy, future studies on double seropositive ON would provide clinical benefit and clarity to a rather unique condition. 

## Conclusions

Bilateral optic neuritis is an interesting condition that has recently been increasingly recognized and better characterized. The discovery of the immune markers AQP-4 and MOG antibodies has revolutionized the approach to these patients. Treatment algorithms are being refined and defined. It is common practice to refer to optic neuritis, especially bilateral optic neuritis, as NMO-positive, MOG-positive, double-positive or double-negative. The arsenal of therapeutics includes IV steroids, intravenous immunoglobulin, plasma exchange and surprisingly oral steroids, as in our case. Our case highlights the unexpected benefit of oral steroids and shows that testing for these autoimmune markers is a prerequisite in the work-up of patients with optic neuritis. As the data and research continue to unfold, we will be better positioned to develop treatment algorithms.
